# Circular RNA circACAP2 Suppresses Ferroptosis of Cervical Cancer during Malignant Progression by miR-193a-5p/GPX4

**DOI:** 10.1155/2022/5228874

**Published:** 2022-07-08

**Authors:** Yingchun Liu, Li Li, Zhe Yang, Dan Wen, Zhenyu Hu

**Affiliations:** ^1^The Second Affiliated Hospital, Hengyang Medical School, University of South China, Hengyang, China; ^2^Affiliated Nanhua Hospital, University of South China, Hengyang, China; ^3^Hengyang Maternal and Child Health Care Hospital, Hengyang, China; ^4^Department of Gynaecology and Obestetrics, TheAffiliated Nanhua Hospital, Hengyang Medical School, University of South China, Hengyang, China

## Abstract

Cervical cancer is among the most cancer types, with an extremely high global incidence and mortality. Ferroptosis is a newly reported programmed cell death process that differs from apoptosis, autophagy, and necroptosis. Circular RNAs (circRNAs) are covalently closed loops generated from back-splicing pre-mRNAs, with high stability, and are abundant in the physical environment. Here, we explored the effect of circACAP2 on ferroptosis of cervical cancer. We observed that the depletion of circACAP2 by siRNAs was validated in cervical cancer cells. The cervical cancer cell viability was inhibited by circACAP2 knockdown as well. The levels of lipid ROS, iron, and Fe^2+^ were reduced by circACAP2 depletion in cervical cancer cells. The circACAP2 served as a ceRNA of miR-193a-5p and directly interacted with miR-193a-5p in cervical cancer cells. miR-193a-5p was able to target GPX4 and circACAP2 promoted GPX4 expression by sponging miR-193a-5p in cervical cancer cells. The knockdown of circACAP2 inhibited the cervical cancer cell viability, but the miR-193a-5p inhibitor or GPX4 overexpression could reverse the effect in the cells. The inhibition of miR-193a-5p or GPX4 overexpression repressed the circACAP2 depletion-induced levels of lipid ROS, iron, and Fe^2+^ in cervical cancer cells. Clinically, the expression of circACAP2 and GPX4 was upregulated, and miR-193a-5p expression was downregulated in clinical cervical cancer samples. The expression of miR-193a-5p was negatively correlated with circACAP2 and GPX4, while the circACAP2 expression was positively correlated with GPX4 in the samples. Therefore, we concluded that circular RNA circACAP2 repressed ferroptosis of cervical cancer during malignant progression by miR-193a-5p/GPX4.

## 1. Introduction

Cervical cancer belongs to the most malignant cancer type with an extremely high incidence and mortality globally [1, 2]. According to the global cancer statistics of GLOBOCAN, there are 604,127 new cases and 341,831 new deaths occurred all over the world in 2020 [3]. Although various therapeutic approaches such as surgery, chemotherapy, and radiotherapy have been applied in clinics, the effective treatment methods for cervical cancer patients are still inadequate [4, 5]. The combined chemotherapy and targeted therapy with bevacizumab, carboplatin, and paclitaxel is a standard treatment for cervical cancer and has notably improved the survival of patients compared with treatment with platinum alone [6]. However, patients with cervical cancer commonly show poor prognosis and low overall survival rate caused by developed drug resistance, metastasis, and relapse [7]. Thus, exploration of novel therapeutic targets and therapies has become imperative and prevalent.

Ferroptosis is a newly reported programmed cell death process that differs from apoptosis, autophagy, and necroptosis, at both morphological and biochemical levels. Ferroptosis is a special process characterized by a lipid peroxidation accumulation in an iron-dependent way, which leads to accumulated reactive oxygen species (ROS) in cells and consequently oxidative cell death [8, 9]. Glutathione (GSH) catalyzed the conversion of accumulated lipid hydroperoxides to lipid alcohols, which is usually regulated by GPX4 and therefore repressing the ferroptosis [10, 11]. On the other hand, when the GSH biosynthesis is interrupted and lipid peroxidation products accumulate, ferroptosis occurs [12]. Noteworthy, compounds such as erastin and RSL3 have been proved as inducers of ferroptosis and are widely applied in basic studies [13]. Accumulating evidence has indicated that aberrant ferroptosis is correlated with malignant cancer progression [14, 15]. Therefore, targeting ferroptosis has become a prevalent research area for cancer treatment. Circular RNAs (circRNAs) are covalently closed loops that generate from back-splicing of pre-mRNAs, with high stability and are abundant in physical environment [16]. circRNAs could directly interact with gene-binding proteins to modulate gene expression and sponge microRNAs (miRNAs) to enhance mRNA stability [17, 18]. The abnormal regulation of circRNAs is significantly correlated with multiple diseases, including cancers [19].

In this work, we explored the role of circACAP2 in cervical cancer and determined that circACAP2 regulated cell ferroptosis through the miR-193a-5p/GPX4 network. Our findings presented circACAP2 as a novel and effective target for cervical cancer treatment.

## 2. Materials and Methods

### 2.1. Specimen Collection

The study has obtained the permission of Ethics Committee of Affiliated Nanhua Hospital, University of South China, no. 2018-171-193. Patients with cervical cancer were recruited from January 2020 to March 2021 and have signed the informed consents. The tumor tissues and adjacent normal tissues were obtained during surgery and stored at −80°C for use. The correlation between circACAP2, miR-193a-5p, and GPX4 was analyzed by Pearson correlation analysis.

### 2.2. Cell Culture

Cervical cancer cells SiHa and HeLa (ATCC, USA) were incubated in a 37°C humidified conditions with 5% CO_2_. Cells were cultured in DMEM (HyClone, USA) that contains 10% fetal bovine serum (FBS; Gibco, USA) and 100 U/ml penicillin and 100 *μ*g/ml streptomycin (Gibco, USA).

### 2.3. Cell Transfection

The cell transfection was conducted using Lipofectamine 3000 (Invitrogen, USA). The miR-193a-5p mimics, miR-193a-5p inhibitors, circACAP2 siRNAs, negative control (shNC), and GPX4 overexpressing vectors were obtained from GenePharma (China). The sequences are as follows: miR-193a-5p mimics: 5′-UGGGUCUUUGCGGGCGAGAUGA-3′; miR-193a-5p inhibitors: 5′-UCAUCUCGCCCGCAAAGACCCA-3; circACAP2 siRNA-1: 5′-GGCAGCATACAGGAAGATGAA-3′; circACAP2 siRNA-2: 5′-UCACCGACUCCCCGGCCGUUU-3′.

### 2.4. Cell Counting Kit-8 (CCK-8) Assay

The cell growth of SiHa and HeLa were measured by the CCK-8 kit (SolarBio, China). The cells (5000 cells each well) transfected with indicated oligonucleotides were planted into a 96-well plate. After incubation for 24, 48, 72, and 96 hours, CCK-8 reagent (10 *μ*l) was added into each well and incubated for 2 hours. Then, absorbance at 450 nm was measured.

### 2.5. RNA Extraction and Quantification

The extraction of total RNA from cervical cancer tissues and cells was realized by using TRIzol (Thermo, USA) in accordance with the manufacturer's instructions. The cDNAs were synthesized by using cDNA synthesis kits (Thermo, USA). The quantitative PCR was conducted using SYBR Green Master Mix (Thermo, USA). Relative mRNA levels were calculated using the 2^−ΔΔCt^ method and normalized to U6 or GAPDH.

The primer sequences are shown (Table 1).

### 2.6. Western Blotting

Whole proteins were obtained by using chilled RIPA lysis buffer (Thermo) followed by quantification with the BCA kit (Thermo). An equal amount of proteins (30 *μ*g) were loaded and separated on the SDS-PAGE gel and blotted to NC membranes (Millipore, USA). The blots were hatched at 4°C overnight with primary antibodies against GPX4 (Abcam, USA), SLC7A11 (Abcam, USA), COX-2 (Abcam, USA), ACSL4 (Abcam, USA), and PTGS2 (Abcam, USA). After that, the blots were incubated with anti-mouse or anti-rabbit secondary antibodies at room temperature for 1 hour. ECL substrate (Thermo, USA) was adopted for visualization of proteins.

### 2.7. Luciferase Reporter Gene Assay

Wild type and mutated fragments of GPX4 3′UTR and circACAP2 were inserted into the pGLO vector (Promega, USA). Cancer cells were transfected with the vectors and seeded into 12-well plates. Cells were harvested, and luciferase intensity was measured using a luciferase reporter gene assay kit (Promega, USA).

### 2.8. RNA Pull-Down Assay

The interaction between circACAP2 and miR-193a-5p was measured by a Pierce™ Magnetic Pull-Down Kit (Thermo, USA) following the manufacturer's protocol. The biotin-labeled miR-193a-5p probe and the negative control were synthesized by RiboBio (China).

### 2.9. Detection of Ferroptosis

The accumulated lipid ROS and iron level were measured by C11-BODIPY (Thermo, USA) and iron assay kit (Abcam, USA) as per manufacturers' protocols.

### 2.10. Statistics

Data in this work were shown as mean ± standard deviation (SD) and statistical analyses were performed using GraphPad Prism 7.0. The values of two or more groups were compared using Student' *t*-tests or one-way ANOVA. *P* < 0.05 was set as significant.

## 3. Results

### 3.1. The Depletion of circACAP2 Reduces Proliferation and Enhances Ferroptosis of Cervical Cancer Cells

**Figure 1:**
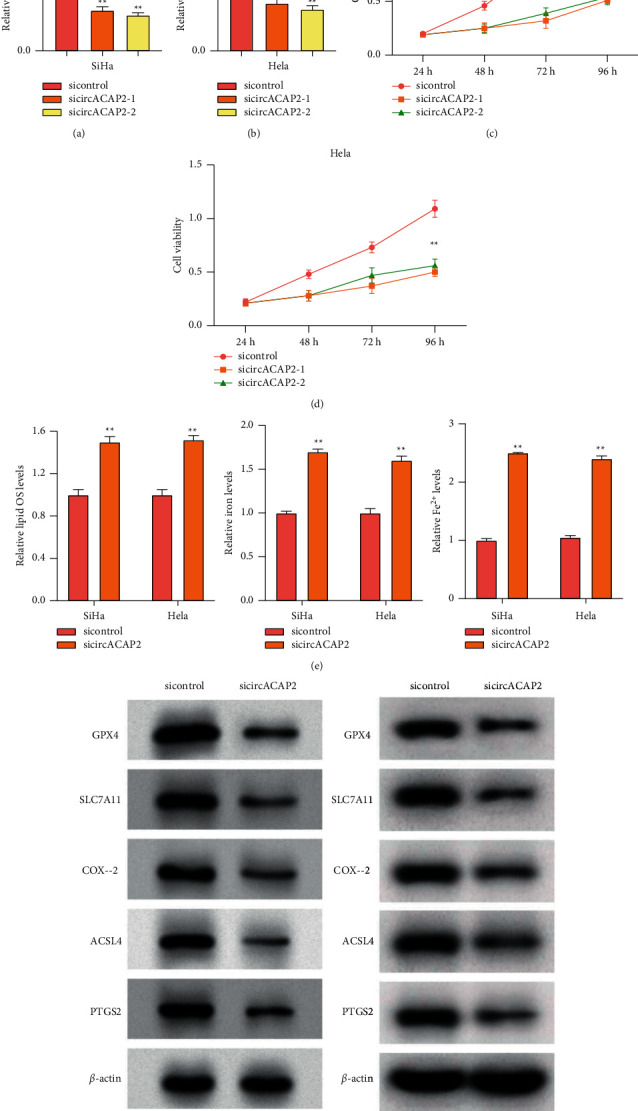
The depletion of circACAP2 reduces proliferation and enhances ferroptosis of cervical cancer cells. (a)–(e) The SiHa and HeLa cells treated with circACAP2 siRNAs. (a), (b) The expression of circACAP2 analyzed by qPCR. (c), (d) MTS assays in the cells. (e) The levels of lipid ROS, iron, and Fe^2+^ detected. (f) The expression of GPX4, SLC7A11, COX-2, ACSL4, and PTGS2 measured by Western blot analysis. ^*∗∗*^*P* < 0.01.

We first assessed the function of circACAP2 in the regulation of proliferation and ferroptosis of cervical cancer cells. The depletion of circACAP2 by siRNAs was validated in SiHa and HeLa cells (Figures 1(a) and 1(b)). The SiHa and HeLa cell viability was inhibited by circACAP2 knockdown as well (Figures 1(c) and 1(d)). In addition, the levels of lipid ROS, iron, and Fe^2+^ were reduced by circACAP2 depletion in SiHa and HeLa cells (Figure 1(e)). Meanwhile, the GPX4 and SLC7A11 expressions were inhibited and COX-2, ACSL4, and PTGS2 expressions were promoted by the depletion of circACAP2 in SiHa and HeLa cells (Figure 1(f)).

### 3.2. CircACAP2 Serves as a ceRNA of miR-193a-5p in Cervical Cancer Cells

**Figure 2:**
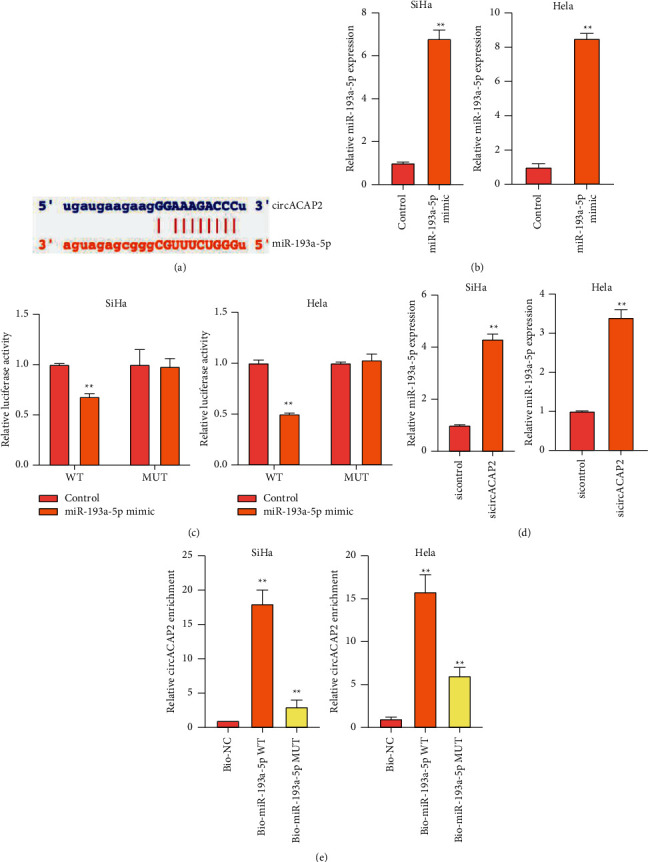
CircACAP2 serves as a ceRNA of miR-193a-5p in cervical cancer cells. (a) The predicted interaction site between circACAP2 and miR-193a-5p. (b), (c) SiHa and HeLa cells treated with miR-193a-5p mimic. The treatment efficacy of miR-193a-5p mimic was determined by qRT-PCR assay. (c) The luciferase activity of circACAP2 analyzed by dual luciferase reporter assay. (d) SiHa and HeLa cells treated with circACAP2 siRNA. The expression of miR-193a-5p was examined by qRT-PCR assay. (e) The interaction of circACAP2 and miR-193a-5p measured by RNA pull-down assay. ^*∗∗*^*P* < 0.01.

We then explored the mechanism of circACAP2-regulated cervical cancer. We found the potential interaction of circACAP2 and miR-193a-5p (Figure 2(a)). The effectiveness of miR-193a-5p mimic was validated in SiHa and HeLa cells (Figure 2(b)). Meanwhile, miR-193a-5p mimic inhibited the luciferase activity of circACAP2 in the cells (Figure 2(c)). The expression of miR-193a-5p was repressed by circACAP2 depletion in SiHa and HeLa cells (Figure 2(d)). In addition, RNA pull-down confirmed the direct interaction of circACAP2 and miR-193a-5p (Figure 2(e)).

### 3.3. MiR-193a-5p Targets GPX4 in Cervical Cancer Cells

**Figure 3:**
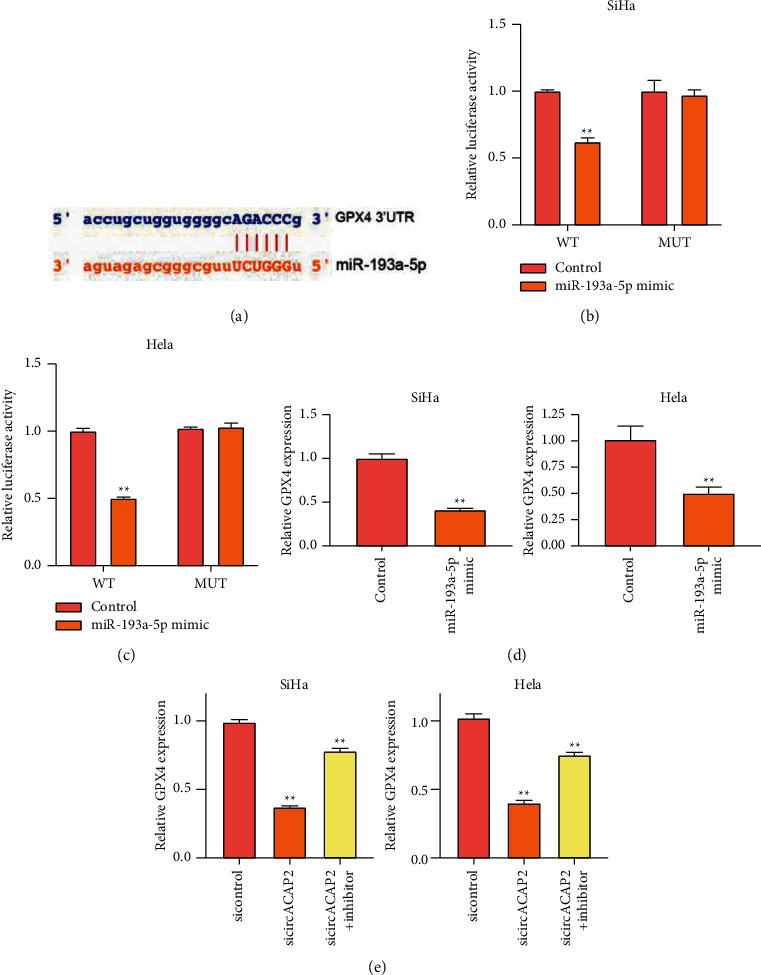
MiR-193a-5p targets GPX4 in cervical cancer cells. (a) The predicted interaction site between miR-193a-5p and GPX4. (b)–(e) SiHa and HeLa cells treated with miR-193a-5p mimic. (b), (c) The luciferase activity of GPX4 determined by dual luciferase reporter assay. (d) The expression of GPX4 measured by qPCR. (e) SiHa and HeLa cells treated with circACAP2 siRNA or cotreated with the miR-193a-5p inhibitor. The expression of GPX4 was analyzed by qPCR. ^*∗∗*^*P* < 0.01.

Next, we found the potential interaction of GPX4 and miR-193a-5p (Figure 3(a)). miR-193a-5p mimic repressed the luciferase activity of GPX4 3′UTR in SiHa and HeLa cells (Figures 3(b) and 3(c)). The expression of GPX4 was inhibited by miR-193a-5p in SiHa and HeLa cells (Figure 3(d)). In addition, the depletion of circACAP2 reduced GPX4 expression, while the inhibitor of miR-193a-5p reversed the reduction in the cells (Figure 3(e)).

### 3.4. CircACAP2 Regulates Proliferation and Ferroptosis by miR-193a-5p/GPX4 Axis Cervical Cancer Cells

**Figure 4:**
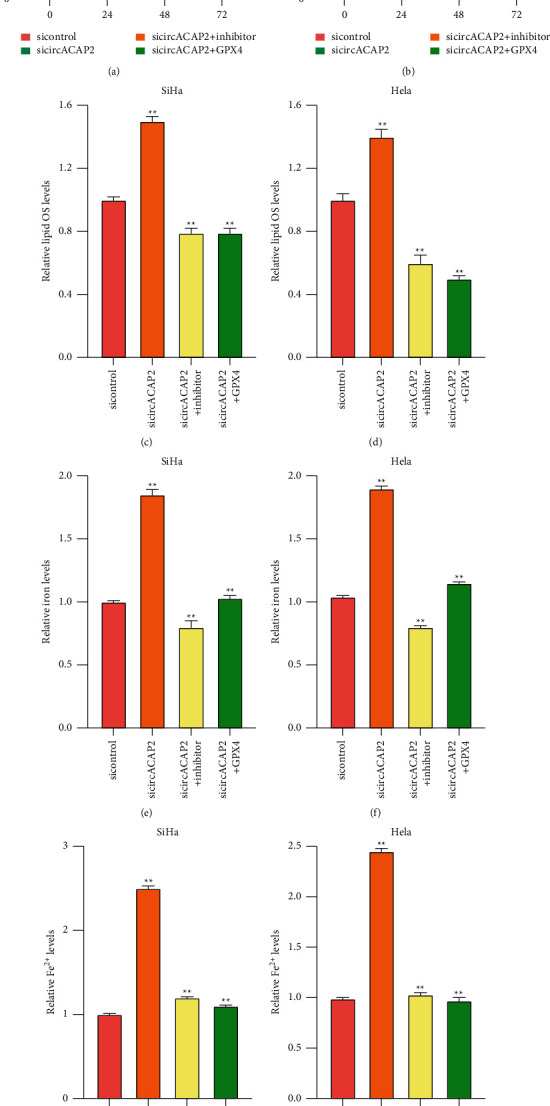
CircACAP2 regulates proliferation and ferroptosis by miR-193a-5p/GPX4 axis cervical cancer cells. (a)–(g) The SiHa and HeLa cells treated with circACAP2 siRNAs or cotreated with GPX4 overexpressing plasmid or miR-193a-5p inhibitor. (a), (b) MTS assays in the cells. (c)–(g) The levels of lipid ROS, iron, and Fe^2+^ detected. ^*∗∗*^*P* < 0.01.

Then, we observed that the knockdown of circACAP2 inhibited the SiHa and HeLa cell viability, but the miR-193a-5p inhibitor or GPX4 overexpression could reverse the effect in the cells (Figures 4(a) and 4(b)). The inhibition of miR-193a-5p or GPX4 overexpression repressed the circACAP2 depletion-induced levels of lipid ROS, iron, and Fe^2+^ in SiHa and HeLa cells (Figures 4(c)–4(g)).

### 3.5. The Clinical Expression and Correlation of circACAP2, miR-193a-5p, and GPX4 in Cervical Cancer

**Figure 5:**
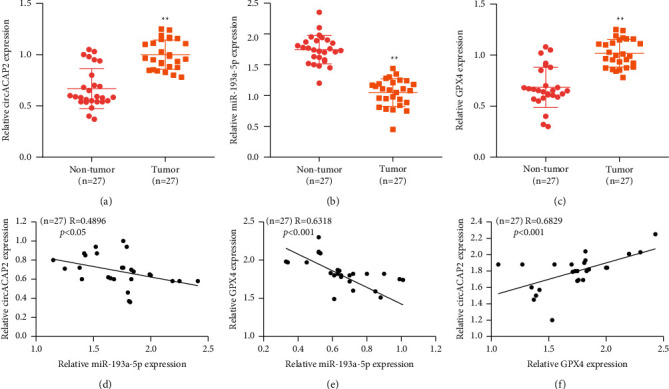
The clinical expression and correlation of circACAP2, miR-193a-5p, and GPX4 in cervical cancer. (a)–(c) The expression of circACAP2, miR-193a-5p, and GPX4 detected by qPCR in clinical cervical cancer samples (*n* = 27). (d)–(f) The correlation of the expression of circACAP2, miR-193a-5p, and GPX4 evaluated (*n* = 27). ^*∗∗*^*P* < 0.01.

We then analyzed the expression and correlation of circACAP2, miR-193a-5p, and GPX4 in cervical cancer samples. We observed that the expression of circACAP2 and GPX4 was upregulated and miR-193a-5p expression was downregulated in clinical cervical cancer samples (Figures 5(a)–5(c)). The expression of miR-193a-5p was negatively correlated with circACAP2 and GPX4, while the circACAP2 expression was positively correlated with GPX4 in the samples (Figures 5(d)–5(f)).

## 4. Discussion

Cervical cancer is the most malignant cancer types with an extremely high incidence and mortality globally. Ferroptosis is a newly reported programmed cell death process that differs from apoptosis, and circRNAs are covalently closed loops that generate from back-splicing of pre-mRNAs, with high stability and are abundant in physical environment. However, the effect of circACAP2 on cervical cancer remains elusive. In this work, we discovered the function of circACAP2 in ferroptosis of cervical cancer.

The circACAP2 plays crucial roles in cancer development. The circRNA-ACAP2 promotes migration and invasion of neuroblastoma cells by miRNA-143-3p/hexokinase 2 signaling [20]. The circACAP2 enhances colorectal cancer progression by miR-143-3p/FZD4 signaling [21]. The circACAP2 contributes to metastasis and proliferation of breast cancer by targeting miR-29a/b-3p-COL5A1 signaling [22]. Our data showed that the depletion of circACAP2 by siRNAs was validated in cervical cancer cells. The cervical cancer cell viability was inhibited by circACAP2 knockdown as well. The levels of lipid ROS, iron, and Fe^2+^ were reduced by circACAP2 depletion in cervical cancer cells. These data provide new evidence of the function of circACAP2 in the modulation of cancer cell ferroptosis, improving the knowledge of the regulatory mechanism of ferroptosis and circRNAs in cervical cancer development. It enriches the understanding of the circACAP2 effect on cervical cancer progression.

Moreover, it has been reported that lncRNA FBXL19-AS1 regulates invasion, migration, and growth of cervical cancer by miR-193a-5p/PIN1 axis [23]. lncRNA FBXL19-AS1 enhances metastasis and proliferation by miR-193a-5p/COL1A1 axis in cervical cancer [24]. The depletion of SFRS9 represses colorectal cancer progression by enhancing ferroptosis by GPX4 [25]. Significantly, we found that circACAP2 served as a ceRNA of miR-193a-5p and directly interacted with miR-193a-5p in cervical cancer cells. miR-193a-5p was able to target GPX4, and circACAP2 promoted the GPX4 expression by sponging miR-193a-5p in cervical cancer cells. The knockdown of circACAP2 inhibited the cervical cancer cell viability, but the miR-193a-5p inhibitor or GPX4 overexpression could reverse the effect in the cells. The inhibition of miR-193a-5p or GPX4 overexpression repressed the circACAP2 depletion-induced levels of lipid ROS, iron, and Fe^2+^ in cervical cancer cells. Clinically, the expression of circACAP2 and GPX4 was upregulated and miR-193a-5p expression was downregulated in clinical cervical cancer samples. The expression of miR-193a-5p was negatively correlated with circACAP2 and GPX4, while the circACAP2 expression was positively correlated with GPX4 in the samples. Our finding provides new insights into the mechanism by which circACAP2 regulates cervical cancer by miR-193a-5p/GPX4.

There are still some limitations in the current study. In this work, we focused on the scientific issue of the effect of circACAP2 on ferroptosis in cervical cancer. The function of circACAP2 in the regulation of other types of programmed cell death such as apoptosis and pyroptosis should be validated in future investigations. Meanwhile, it has been identified that miR-193a-5p plays a critical role in the development of cervical cancer [23, 24], and we identified the potential interaction between circACAP2 and miR-193a-5p in the bioinformatic analysis. However, as we all know, circRNAs target multiple downstream miRNAs and other miRNAs sponged by circACAP2 need to be identified in cervical cancer progression.

We concluded that circular RNA circACAP2 repressed ferroptosis of cervical cancer during malignant progression by miR-193a-5p/GPX4.

## Figures and Tables

**Table 1:** Primer information.

Gene	Primer
circACAP2	5′-GAATGGGATTCGAGACCTG-3′ 5′-TTCTTCCAAAGCTGCCTGT-3′

miR-193a-5p	5′-TATATGGGTCTTTGCGGGCG-3′ 5′-GTGCAGGGTCCGAGGT-3′

GPX4	5′-TTCTCGGACGATGGCTACC-3′ 5′-GAACCAAGTGAGAGACAGAATGACC-3′

GAPDH	5′-AAGAAGGTGGTGAAGCAGGC-3′ 5′-TCCACCACCCAGTTGCTGTA-3′

U6	5′-CTCGCTTCGGCAGCACA-3′ 5′-AACGCTTCACGAATTTGCGT-3′

## Data Availability

The datasets used during the present study are available from the corresponding author upon request.
